# Non-syndromic craniosynostosis in children: Scoping review

**DOI:** 10.4317/medoral.22328

**Published:** 2018-06-21

**Authors:** Arturo Garrocho-Rangel, Lizeth Manríquez-Olmos, Joselín Flores-Velázquez, Miguel-Ángel Rosales-Berber, Ricardo Martínez-Rider, Amaury Pozos-Guillén

**Affiliations:** 1 DDS, MSc, PhD, Especialidad en Estomatología Pediátrica, Facultad de Estomatología, Universidad Autónoma de San Luis Potosí, San Luis Potosí, S.L.P., México; 2 DDS, Especialidad en Estomatología Pediátrica, Facultad de Estomatología, Universidad Autónoma de San Luis Potosí, San Luis Potosí, S.L.P., México; 3 DDS, Especialidad en Estomatología Pediátrica, Facultad de Estomatología, Universidad Autónoma de San Luis Potosí, San Luis Potosí, S.L.P., México; 4 DDS, MSc, Especialidad en Estomatología Pediátrica, Facultad de Estomatología, Universidad Autónoma de San Luis Potosí, San Luis Potosí, S.L.P., México; 5 DDS, Especialidad en Estomatología Pediátrica, Facultad de Estomatología, Universidad Autónoma de San Luis Potosí, San Luis Potosí, S.L.P., México; 6 DDS, MSc, PhD, Laboratorio de Ciencias Básicas, Facultad de Estomatología, Universidad Autónoma de San Luis Potosí, San Luis Potosí, S.L.P., México

## Abstract

**Background:**

Craniosynostosis (CS) is a complex condition consisting of the early fusion of one or more cranial sutures in the intrauterine stage. The affected infant exhibits abnormal head shape at time of birth or shortly thereafter. It can be observed in normal individuals (non-syndromic CS or NSCS) or as a part of a multisystem syndrome. The purposes of the present article were to carry out a scoping review on Non-Syndromic CS and to discuss the most important findings retrieved.

**Material and Methods:**

The steps of this scoping review were as follows: first, to pose a research question; second, to identify relevant studies to answer the research question; third, to select and retrieve the studies; fourth, to chart the critical data, and finally, to collate, summarize, and report the results from the most important articles. Relevant articles published over a 20-year period were identified and retrieved from five Internet databases: PubMed; EMBASE; Cochrane Library; Google Scholar, and EBSCO.

**Results:**

Fourteen articles were finally included in the present scoping review. The following four most important clinical issues are discussed: (i) normal cranial development, clinical manifestations, and pathogenesis of NCSC; (ii) clinical evaluation of NCSC; (iii) treatment and post-surgical follow-up; and (iv) additional considerations.

**Conclusions:**

NSCS may be present with associated head shapes. Multiple early surgical reconstructive options are currently available for the disorder. Pediatric Dentistry practitioners must be familiarized with this condition and form part of a multi-approach health team as those responsible for the opportune oral health care of the affected child.

** Key words:**Craniosynostosis, cranial development, children, scoping review, dental management.

## Introduction

Children born with craniofacial anomalies exhibit multiple and complex problems when performing different physiologic functions, such as early feeding, hearing, and speech. Additionally, these conditions are strongly associated with dentofacial/occlusal abnormalities and can interfere in the psychosocial adjustment process (1).

Craniosynostosis (CS) refers to the premature fusion in the perinatal stage of one or multiple skull sutures, also denominated synostoses (sagittal, metopic, uni- and bilateral coronal, and lamboidal), which are commonly accompanied by facial, trunk, and limb deformities (2-5). This condition may occur either in an isolated way –representing about 85% of cases and called Non-Syndromic Craniosynostosis (NSCS)–, or associated with more than 150 different structural malformations or syndromes (known as syndromic craniosynostoses) (6). Some commonly cited CS syndromes include Apert, Crouzon, Pfeiffer, Muenke, Saethre-Chotzen, and Antley-Bixter (7,8).

CS was first described by the German Surgeon Samuel Thomas von Soemmerring in 1791 (9). In 1851, Virchow coined the term “craniosynostosis” and hypothesized that the phenomenon was the consequence of cretinism or meningeal inflammation, while Moss (1959) thought that the cranial base was the source of the condition (4,10). The incidence of CS has been estimated at 1 per 2,000-2,500 live newborns, thus comprising the second most common craniofacial disorder after orofacial clefts (5,11,12). However, some of the malformations result in fetal death (13). Approximately 80% of cases belong to the NSCS group (8). CS occurs more commonly overall in boys than in girls (5). There is no predilection for a specific geographic region, ethnic group, or socioeconomic status (11). Diverse anomalies may be present in patients with CS, such as cleft soft palate, hypodontia or hyperodontia, delayed tooth eruption, taurodontism, microdontia, multiple dens invaginatus, and dentin dysplasia (2). Diagnosis of CS in infants is based on the observation of an abnormal head shape, together clinical and image (x-ray and three-dimensional [3D] Computed Tomography [CT] scanning) assessments, together with 3D soft and bone tissue reconstructions (3,12,13). Management of CS should be assumed by a multidisciplinary craniofacial team composed of different health specialists in which the Pediatric Dentist must be involved, with the common purpose of providing a comprehensive medical care (3,6,10,14,15). Treatment of this disorder is mainly surgical, consisting of the excision of fused sutures prior to 12 months of age.

In this context, it is important for Pediatric Dentists to be familiarized with the nuances of CS and to know its main clinical presentations. Thus, the main purposes of the present article were to carry out a scoping review of the most relevant literature on CS and to discuss the most important findings collected during this process.

## Material and Methods

The present scoping review was carried out in accordance with guidelines for reporting the scoping review (16,17). This framework includes five steps as follows: (i) designing the research question; (ii) identifying relevant studies through a search of the literature; (iii) study selection; (iv) data extraction and charting, and (v) collating, summarizing, and reporting the results.

-Research question 

A research question was structured based on the PICO format (Patient/Intervention/Comparison/Outcome) to scope the extent of research available on the clinical topic (NSCS) during the search process: What are the principal oral health care necessities of children with Non-Syndromic Craniosynostosis (NSCS)?

-Identification of relevant studies

To find potentially relevant articles, the following electronic databases were exhaustively searched: PubMed; EMBASE/Ovid; Cochrane Library; Google Scholar, and EBSCO (Dentistry& Oral Science Source) during September, 2017. Only references published over the last 20 years (July 1997 to September 2017) whose purpose it was to identify the potential clinical needs of pediatric patients with NSCS, were screened. To be eligible for review, articles were required to meet the following criteria: randomized clinical trials; observational studies (cohorts, case-control designs, cross-sectional studies, and clinical case reports), or review articles; written in English or Spanish, and focused on infants and children with NSCS. Gray literature, comments, editorials, short communications, and letters were excluded.

A comprehensive literature search (electronic and manual) was independently conducted by three authors (LM-O, JF-V, MN-F) in order to identify appropriate titles and abstracts. A search strategy was carefully implemented, using three major concepts: “non-syndromic craniosynostosis”; “infants and children”, and “oral health care”. Several search/MeSh terms, keywords, or synonyms were combined and appropriately adapted for each database. Then, chosen articles were retrieved in full-text and read and assessed by other two experienced reviewers (JAG-R and AJP-G) separately for the final list of the studies included. The reference lists on which each selected article appeared were also screened to discern other potential eligible studies. Any discrepancy was discussed and resolved by consensus with the aid of a third examiner (RM-R).

-Data extraction

Data from eligible studies were extracted and entered into a pre-designed and piloted standardized tracking and review form in order to present a narrative account of the relevant literature and to avoid overlapping. From each individual article, the following information was extracted and recorded: general characteristics (authors, year of publication, methodological design, and study setting); the patients’ clinical features (age, gender, medical condition, type of NSCS, oral status, etc.), oral management (e.g., diagnostic methods, oral hygiene/preventive management, behavioral issues, and treatment procedures), measured main outcome, key findings or conclusions, and authors’ recommendations. A judgment concerning whether each outcome was primarily clinician-centered was also performed. Thereafter, data were collected, detailed, cross-checked, summarized (in tables or charts), and discussed accordingly. Additionally, the scoping review process was structured in the form of a flow diagram, according to PRISMA guidelines (18).

## Results

**Figure 1:**
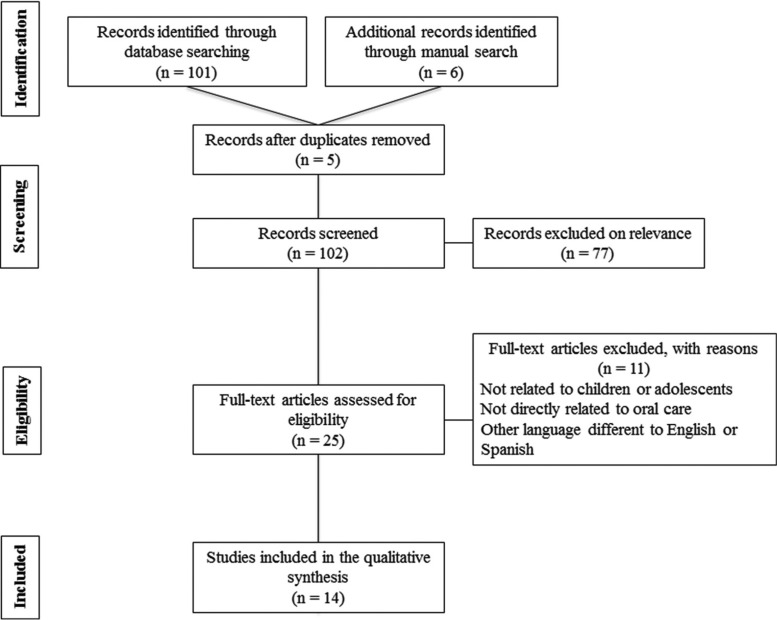
PRISMA flow diagram of literature search.

We identified 107 references of potentially relevant articles. Following duplicate removal (n = 5), 102 articles were detail-screened, and 25 of them were selected for full-text review. Of these, 14 studies published between 1997 and 2017 were finally included in the present scoping review. The whole selection process is described in the flow diagram of Figure 1. Additionally,  Table 1,  Table 1 continue,  Table 1-1 continue presents the general characteristics of the studies included in this scoping review. The majority of the articles were narrative reviews/guidelines; only three publications were original investigations, including one retrospective cohort study (9), one case-control study (6), and one descriptive/exploratory study (15). Only one study (2) mentions in detail the main oral features/dental manifestations reported in pediatric patients with NSCS. After exploring the final selection of studies, a large amount of relevant clinical information was condensed. The main findings deriving from this process are enlisted in the Discussion section.

**Table 1:**
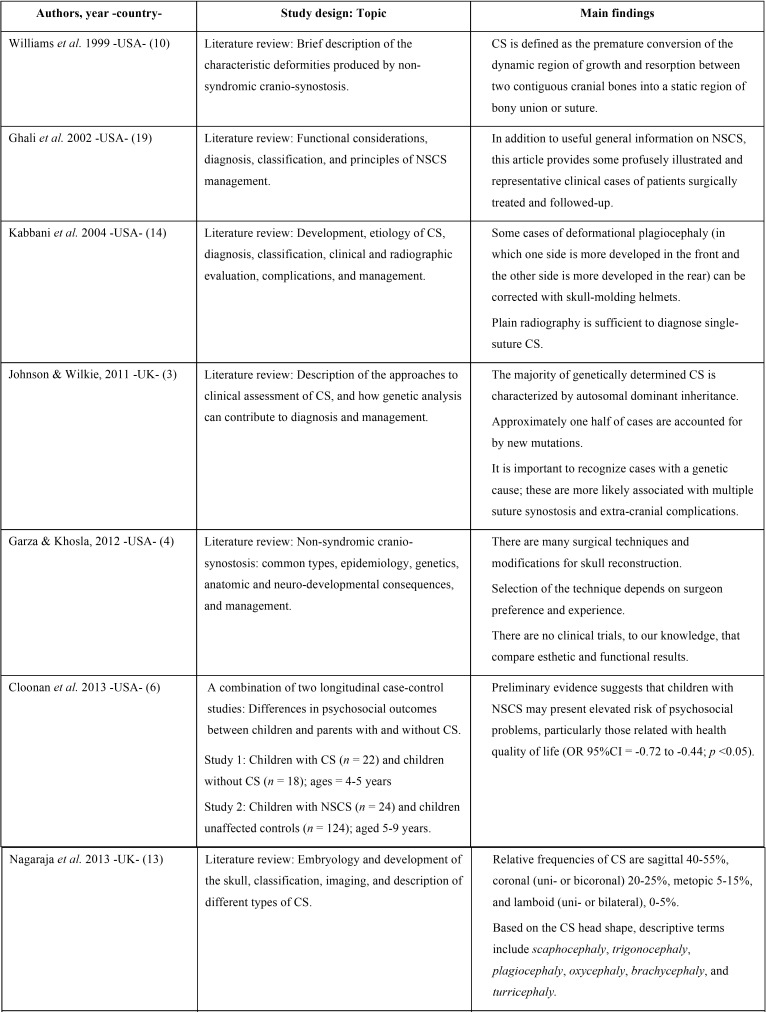
List of the 14 studies included in the present scoping review and their general characteristics.

**Table 1 continue:**
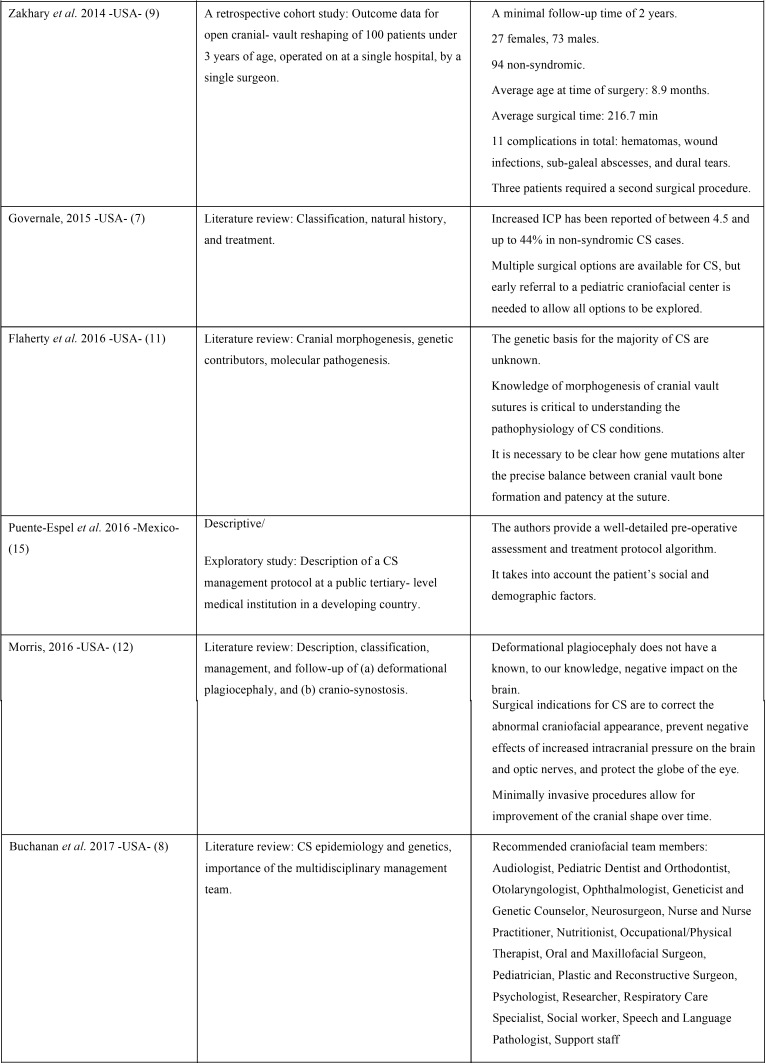
List of the 14 studies included in the present scoping review and their general characteristics.

**Table 1-1 continue:**
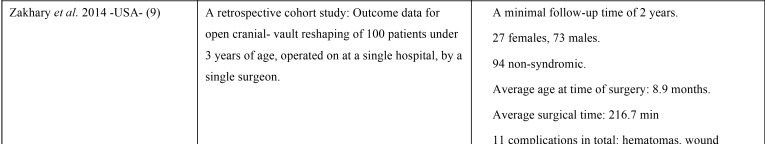
List of the 14 studies included in the present scoping review and their general characteristics.

## Discussion

After reviewing the main findings of the present scoping review, three relevant clinical topics regarding NSCS were considered of greatest interest in terms of the Pediatric Dentistry practice: (i) normal cranial development and pathogenesis of NCSC; (ii) clinical evaluation of NCSC; (iii) treatment of NSCS and post-surgical follow-up, and (iv) additional considerations. The following discussion will be focused on the following four matters.

i. Normal cranial development, clinical manifestations, and pathogenesis of NCSC. During normal human body and head development, cranial growth achieves approximately 80% of the adult size at birth and its definitive size between 2.5 and 3 years of age. In the fetal or newborn skull, the flat bones are separated by four fontanelles and six major cranial sutures that participate in this process (5,14). The paired frontal and parietal bones are separated at the midline by the metopic and sagittal sutures, respectively, the frontal and parietal bones are separated by the coronal sutures, and the parietal bones are separated from the single occipital bone by the lamboid sutures (3). Each suture is composed by a dense fibrous connection that separates the individual cranial bones. Sutures allow for physiological skull expansion and also for transitory transvaginal (or birth canal) head compression, during birth (12,19). Under normal circumstances, the sutures and fontanelles close at different times during life: from the age of 3 months of age until the third decade of life and even beyond (14).

However, and as a consequence of premature fusion of the calvarial suture, skull growth is restricted parallel to the affected suture; in addition, the growing brain beneath the suture is limited as well, due to the inability of the involved sutures to accommodate this structure (8). In other words, distortion of the skull shape is primarily due to a combination of lack of growth perpendicular to the fused suture, and compensatory overgrowth at the non-fused sutures (3). These conditions lead to compensatory brain expansion into regions of the cranial vault that are not affected by CS, causing a cranial progressive deformity (5,12). Thus, there are different types of NSCS-characteristic dysmorphic head shapes with specific clinical findings, depending on the number of sutures fused and the involved regions (12,15). Single suture synostosis most frequently affects the sagittal suture, followed by the coronal, metopic, and lamboid sutures (3,14).

The pathogenesis of CS is unclear, complex, and perhaps multifactorial, including intrinsic bone abnormalities, genetic mutations, and environmental (mechanical or biochemical) issues (3,4,12,13,19). CS has been associated with metabolic conditions (hypophosphatemia, rickets), and with other risk factors as follows: fetal constraint (nulliparity, plurality, macrosomia); low birth weight; hyperthyroidism; maternal smoking; pre-term delivery; exposure to teratogens; maternal consumption of valproate acid; shunted hydrocephalus, and excessive ingestion of antiacids (5,7). A single genetic anomaly has not been identified as a causal factor for the condition. The genes most frequently involved in CS include those encoding for the different fibroblast growth-factor receptors (3); these mutations lead to defects in signaling and tissue interactions, resulting in abnormal suture maturation and cranial malformation, particularly in the syndromic type (2,3,8,9).

ii. Clinical evaluation of NCSC. The most common clinical presentation of NSCS is an unusual head shape during the first year of life, in which the head may be long and narrow (scaphocephaly and/or dolichocephaly), or triangular at the front (trigonocephaly), or broad and flattened (brachycephaly), or skewed (plagiocephaly) (3). Under these circumstances, the major functional complications associated with the disorder mentioned in the literature comprise intracranial hypertension, visual impairment, limitation of brain growth, hydrocephalus, and neuropsychiatric disorders; these anomalies are often irreversible (7,9,19).

Clinical evaluation consists of palpation of the skull for any movement, ridging, and the presence of fontanelles; sometimes, specific quantitative cranial anthropometric measurements are performed (14,19). It is recommend that the examination follow a set pattern to avoid overlooking clues, starting with the hands and feet, looking for congenital anomalies. NSCS should be differentiated from other craniofacial disorders, for instance, positional plagiocephaly (3,7).

iii. Treatment of NSCS and post-surgical follow-up. If left untreated, NSCS can result in aggravated craniofacial deformities, which may lead to psychosocial issues as the child interacts with peers during development, due to visible facial differences or language/visual/behavior impairments (4,5,7). Affected children may have an increased risk for psychosocial and cognitive difficulties, and consequently, a diminished health-related quality of life (6). In addition, parents are psychologically influenced by the experience of having a child with birth anomaly, for instance, parents exhibit behavioral patterns such as stress due to the surgical procedure, possible infant mortality, and concerns regarding the child’s future. These factors likely affect the care-giving process and the child’s psychosocial adaptation (6).

General management of infants and children with NSCS is directed toward correcting and preventing progression of the skull deformity, stabilizing the elevated intracranial pressure, maintaining the airway, and supporting the feeding, optimal oral health, and eye protection (3). It is suggested that patients be managed in a specialized pediatric craniofacial center with all of the necessary medical/dental staff, technical expertise, resources, and equipment (15). The disorder is usually treated surgically soon after diagnosis to unlock and reshape the bones in order to optimize correction of the craniofacial malformations to reduce the effects of the increased intracranial pressure, and for functional and esthetic reasons (7). For these purposes, minimally invasive techniques have been proposed to reduce surgical morbidity, with significantly less blood loss and shorter hospital stay (14). Currently, there are diverse recommended surgical techniques that include the following: open calvarial reconstruction; strip craniectomy with the use of a post-operative molding helmet; strip craniectomy with spring implantation, endoscopic suture release, and cranial distraction osteogenesis (7,10). Fundamental aspects of the surgical management of different craniosynostoses are described in Table 2. Timing of the surgical procedure has been advocated during the first few weeks after birth or during the first year of life, preferably prior to 9 months of age (14,19). In some severely affected patients, a second surgical intervention is indicated to correct residual deformities (9). Additionally, newly available biomaterials have been recently developed together with recent advances in pediatric anesthesia, for employment in the treatment of children with NSCS, for instance, bone substitutes such as resorbable fixation systems and hydroxyapatite cements (10,19).

In any case, after reparative surgery, patient control and follow-up continue throughout childhood and adolescence until skeletal maturity (4,15). Affected children under 5 years of age are reviewed annually, whereas children over 5 years of age are seen every other year. These frequencies vary with the stability of the deformity and its consequences. At these appointments, patients should be evaluated for signs and symptoms of increased intracranial pressure (e.g., headache, nausea and vomiting, developmental delay, irritability, visual disturbances, declining academic performance, and seizures) and for esthetic results (12,19).

iv. Additional considerations. The American Association of People with Disabilities (AAPD) states that “patients with craniofacial anomalies require dental care throughout life as a direct result of their condition and as an integral part of the treatment process”. In this regard and according to De Coster *et al.*, (2) unlike the syndromic type, little has been reported on oral features and dental manifestations of patients with NSCS. They mention that taurodontism, microdontia, and agenesis have been commonly reported, combined or as a solitary trait, and have been accorded important diagnostic weight; these findings strongly suggest that the same genes, transcription factors, and pathways that cause CS interact and thus may play a key role in the development and morphogenesis of the teeth.

Thus, Pediatric Dentistry practitioners should be responsible for the integral oral care of children affected by NSCS, initiating immediately prior to the eruption of the first primary teeth and no later than when the patient is 12 months of age. This process includes systematic clinical examinations for skeletal and dental components, diagnostic recording, caries and gingival/periodontal control, preventive management, language development, and rehabilitative treatment (e.g., restorative, interceptive orthodontic/orthopedic, and prosthetic). Close cooperation is recommended with other specialists, such as the Maxillofacial Surgeon and the Speech-Language Therapist (1).

## Conclusions

NSCS may continue to be a diagnostic and therapeutic challenge. Early recognition, diagnosis, and proper management of the NSCS should be performed by a competent multidisciplinary medical/dental team, with the common aim of improving the function and the psychological well-being of the patient. Pediatric Dentistry practitioners must be active participants in these teams. In addition, they should always be aware that children affected by NSCS are at higher risk of exhibiting psychosocial sequelae that affect the process of providing adequate oral health care. However, early management of this condition can bring about significant improvements in the patient’s quality of life.
